# What minimal detectable effect size is in your power – An inverted sample size formular for survival data

**DOI:** 10.1192/j.eurpsy.2023.1139

**Published:** 2023-07-19

**Authors:** M. K. Dalgaard, A.-E. Christensen, M. K. Kjeldsen

**Affiliations:** 1 Aalborg University Hospital, Psychiatry, Unit for Psychiatric Research, Aalborg, Denmark

## Abstract

**Introduction:**

Power calculations are widely used in the conduct of clinical trials and are often required in funding applications and approvals. There is a recent debate on the role of power calculations in observational studies on existing data with (Hernán J Clin Epidemiol 2022; 144 203-205) and (Moris and Smeden J Clin Epidemiol 2022; 142 261-263) emphasizing the need for planning for all study types without risking discarding imprecise but otherwise relevant studies. In the current study, we construct a graph useful in the planning of a wide range of studies with survival data. We map the minimal detectable effect (MDE) for any possible number of events with a dichotome exposure varying the proportion assigned to the exposure groups.

**Objectives:**

To provide a visual tool relating the sample size, more precisely the number of events, and the MDE for survival data in unbalanced designs.

**Methods:**

The visualization is based on the formulas used by Stata’s power logrank function by (Schoenfeld Biometrics 1983; 39 499-503) and (Freedman Statistics in medicine 1982; 1 121-129), and the MDE is mapped as a function of the number of events. Furthermore, we apply this to an ongoing project on data from the Danish national registers, comparing the risk of developing polycystic ovary syndrome (PCOS) associated with treatment with valproic acid in a population with bipolar disorder or epilepsy.

**Results:**

Preliminary results (Fig. 1) show, as expected, that a larger sample size is required to obtain an MDE close to one. Also, the MDE increases when the assignment among groups is skewed. Moreover, we find a relevant minimal detectable HRR of 1.78 for developing PCOS in a population of 13,839 patients with bipolar disorder or epilepsy, exposed to valproic acid versus those not exposed to valproic acid, with a total of 203 cases of PCOS.

Fig. 1 shows the minimal detectable effect size as a function of the number of events, with α = 0.05 and a power of 1 - β = 0.8, for various assignment ratios φ = P_T_/P_C_, where P_T_ and P_C_ are the proportions of patients assigned to the treatment group and the control group, respectively.

**Image:**

**Figure:**
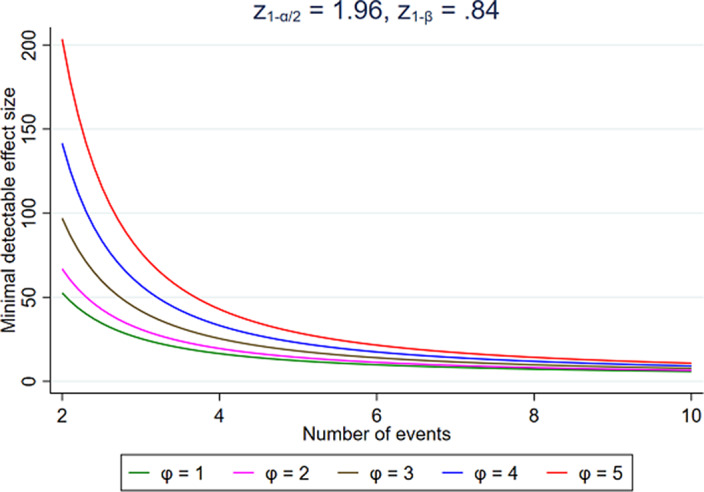

**Conclusions:**

The current visualization and corresponding calculation can be used to guide decisions in the design phase of both observational studies as well as in clinical trials. For observational studies, the sample size, or equivalently, the number of events, could well be fixed, and the MDE may help assess the clinical relevance of conducting the study as in the example with PCOS data. The curves can also provide insight into which efforts might lower the MDE, e.g., whether a small increase in sample size or a different assignment proportion would be most beneficial based on a given sample size.

**Disclosure of Interest:**

None Declared

